# Network modularity influences plant reproduction in a mosaic tropical agroecosystem

**DOI:** 10.1098/rspb.2019.0296

**Published:** 2019-03-27

**Authors:** Manu E. Saunders, Romina Rader

**Affiliations:** 1 School of Environmental and Rural Science, University of New England, Armidale, New South Wales 2351, Australia; 2 UNE Business School, University of New England, Armidale, New South Wales 2351, Australia

**Keywords:** ecosystem function, modularity, ecological networks, plant–pollinator networks, diptera, syrphidae

## Abstract

Biodiversity influences ecosystem function, but there is limited understanding of the mechanisms that support this relationship across different land use types in mosaic agroecosystems. Network approaches can help to understand how community structure influences ecosystem function across landscapes; however, in ecology, network analyses have largely focused on species–species interactions. Here, we use bipartite network analysis in a novel way: to link pollinator communities to sites in a tropical agricultural landscape. We used sentinel plants of *Brassica rapa* to examine how the structure of the community network influences plant reproduction. Diptera was the most common order of flower visitors at every site. Syrphidae visits were the strongest contributor to the number of fertilized pods, while visits by Syrphidae, Hymenoptera and Lepidoptera had the strongest effect on the number of seeds per pod. Sentinel pots at forest sites were visited by more unique species (i.e. species with higher *d′*) than sites in other land uses, and dairy sites had more visitors that were common across the network. Participation coefficients, which indicate how connected a single node is across network modules, were strong predictors of ecosystem function: plant reproduction increased at sites with higher participation coefficients. Flower visitor taxa with higher participation coefficients also had the strongest effect on plant reproduction. Hymenoptera visits were the best predictor for participation coefficients but an *Allograpta* sp. (Diptera: Syrphidae) was the most influential flower visitor species in the landscape network. A diverse insect community contributed to plant reproduction and connection among nodes in this system. Identifying the ‘keystone’ flower visitor species and sites that have a strong influence on network structure is a significant step forward to inform conservation priorities and decision-making in diverse agroecosystems.

## 1. Introduction

Biodiversity is a key driver of ecosystem function, but a greater understanding of the mechanisms that drive relationships between ecological community structure and ecosystem function is needed to inform sustainable land management [1,2]. Pollination by mobile organisms is essential to the provision of ecosystem services in natural and modified habitats [3], yet pollination services are under threat from multiple anthropogenic pressures, including many stressors associated with land use intensification and modification [4]. The expansion of agriculture is a major driver of land use change, often resulting in a mosaic of different natural and anthropogenic land uses [5]. These land use changes can affect the population dynamics of insect pollinators, in turn impacting their functional role in plant reproduction. However, the extent to which land use change has positive or negative effects on pollinator communities and associated pollination services depends on the context, including characteristics of land use and the ecology of interacting species [3,6].

Pollinator assemblages in agricultural landscapes are often very different to those in less disturbed systems [7]. Changing land use affects the abundance and richness of some pollinator taxa in agricultural landscapes, resulting in ‘winner’ and ‘loser’ species (e.g. [8–10]). Yet diverse pollinator communities, often with many common generalist species, are essential to enhance production in multiple types of agroecosystems at different scales [11–14]. Therefore, we need more studies that build an understanding of community-level interactions across land use types and develop methods for linking community structure (e.g. composition and interactions) with ecosystem function [15].

Network approaches provide promising methods to advance understanding of these relationships. Historically, studies of plant–pollinator interactions focused on highly specialized coevolved species, predominantly in natural systems with limited human impacts, and network approaches have been adopted only recently to shift the focus to community-level dynamics [16]. However, much of this work is descriptive and there is still limited understanding of how plant–pollinator network structure affects ecosystem function, or associated ecosystem services (e.g. fruit/seed production). Recent work has shown that plant fitness may be related to the plant's position in the network (e.g. [17]), but the relationship between whole-network structure and functional outcomes (e.g. pollination success) requires further investigation [15,18]. Network structure is influenced by landscape complexity and composition [19,20], and emerging evidence suggests links between network structure and ecosystem function [21]. Yet it is still unclear how network interactions and associated energy flows are partitioned across landscapes and habitats. Spatially explicit analyses that link spatial connectivity of networks with ecosystem function are needed to increase understanding of how interactions affect ecosystem stability and resilience [21]. In particular, there is a need for recent theoretical advances in this area to be adapted to solve applied ecology and conservation problems, especially relevant to ecosystem services [15,22].

Network analysis in community ecology has largely focused on food webs, and host–parasitoid and plant–pollinator networks [23–25]. More recently, the utility of traditional network approaches to solve conservation and applied ecology problems has been demonstrated [22,26,27]. Greater understanding of how the structure of interaction networks influences ecosystem function, as well as how landscape management influences network structure, is essential to inform decisions that sustain biodiversity and ecosystem services in managed landscapes [22]. Historically, analyses of network structure have largely concentrated on global properties, such as degree distribution. However, connectivity metrics, like modularity, may be more informative for understanding functional properties [28]. Modularity focuses on the complementarity of species in different modules (i.e. species may be redundant within their own module but act as complements between modules [29]). Complementarity is an important mechanism to identify in pollination networks, because diverse pollinator assemblages buffer ecosystem function (and associated services) from temporal dynamics and climatic change [30–34]. The level of specialization in pollination networks is also informative because, while common or abundant pollinator species may be important for providing pollination services in many systems, relatively rare and specialized species are equally important across different spatial and temporal scales [14].

Here, we investigate how land use type within an agricultural matrix influences the structure of plant–pollinator networks and the provision of ecosystem services. We use a dataset of insect pollination of sentinel pots of a generalist crop plant, *Brassica rapa* L., collected in four land use types (dairy, rotational cropping, avocado orchard and remnant forest) across a tropical agricultural landscape in north Queensland, Australia. We use a novel systems approach, bipartite site–pollinator (cf. plant–pollinator) networks, to test whether the structure of pollinator communities is linked to ecosystem function at the landscape scale. Specifically, we ask the following questions:

1. How does land use type influence the composition and flower visitation of insect pollinator orders and sentinel plant reproduction?
2. How does land use type and distance between sites influence node specialization (*d′*) and modularity of the bipartite site–pollinator species network?
3. Does node specialization (*d′*) and modularity of the site–pollinator species network influence sentinel plant reproduction?

## 2. Material and methods

### (a) Field data collection

We collected data on flower visitation and plant reproduction for sentinel pots of *B. rapa* ssp. *chinensis* at 20 sites (electronic supplementary material, figure S1) in a tropical agricultural landscape in the Atherton Tablelands (17°18′ S, 145°29′ E to 17°36′ S, 145°44′ E), northeast Australia (data collected by RR from April to May 2008, as part of a larger study of pollinator communities in the study region from 2008 to 2010). *B. rapa* ssp. *chinensis* was chosen as a suitable sentinel plant study species, as it is self-incompatible [35], easy to grow, flowers within 30–50 days, has been used as a sentinel species in other systems and is a generalist species that attracts a wide range of insect pollinators [36]. As insect pollinators respond to the density of floral resources at the local scale [37,38], this provided some means of standardization at a fine scale to conduct our floral visitation surveys. Sites were selected within four different land uses: remnant forest (*n* = 6), avocado orchards (*n* = 5), dairy pastures (*n* = 4) and rotational potato fields (*n* = 5), which are four of the most valuable and well-established industries in the study region [39]. Each site was at least 1 km away from any other, and most were more than 5 km away. We hypothesize that disturbance intensity (from human management activities) was highest in potato farms (rotational cropping), moderate in dairy pastures, medium in avocado orchards and low in the remnant forest.

Sentinel pots were germinated at CSIRO Atherton and placed in the field at budding stage, arranged together as a potted flower ‘island’ at the site in an open area for maximum light availability. All sites had 15–18 individual budding plants and all plants were watered every 3–5 days. All observations were carried out between 10.00 and 16.00 on sunny or partly cloudy days within a temperature range of 16–25°C during peak flowering of potted plants. Observations were not conducted if it was raining or if wind speed was greater than 5 m s^−1^. Six rotations of site observations were conducted during the sampling period. Sites were chosen at random in each sampling rotation so that each observation per site was conducted on different days at different times of the day (i.e. independent replicates). Each full rotation took approximately 2–4 days, depending on the weather. Flower visitation frequency of insect taxa was measured by observing potted ‘islands’ at each site and recording species and number of visits of all insect visitors that landed on any potted flower during six 30 min observation periods at each site. Individuals of insect species that could not be identified in the field were collected (outside of the observation period) and returned to the laboratory for identification. All insects were identified to species or morphospecies; hereafter, we simply use the term species.

Approximately 60 days after first flowering, pots were returned to the shade-house and the following plant attributes were recorded: the number of inflorescences per plant; the number of flowers per inflorescence that were not fertilized (these were identified by withered pedicels); the number of fertilized seed pods per inflorescence; the number of seeds within each seed pod. As the number of flowers varied among plants and sites, individual plant reproductive success was standardized for the number of open flowers available to pollinators, subsequently measured as: (i) the proportion of fertilized seed pods, defined as the number of seed pods per plant/(number of unfertilized flowers + number of seed pods per plant); and (ii) the number of seeds in each pod. Hereafter, we call these ‘fertilized pods’ and ‘number of seeds per pod’ respectively.

### (b) Statistical analysis

To assess differences in the community composition of taxonomic orders among land use types (question 1), we used non-metric multi-dimensional scaling (NMDS) and a PERMANOVA test (both based on Bray–Curtis similarities). We used a generalized linear model with a quasi-Poisson error structure (to account for overdispersion) to compare differences between total visits for each taxonomic order: Diptera, Hymenoptera, Coleoptera and Lepidoptera. We removed Hemiptera from this analysis, because this order was represented by only eight individuals observed at one avocado site (see supplementary data under ‘Data accessibility’ below). We used generalized linear models (GLMs) to identify differences between land use types for the following variables: species richness of flower visitors; total number of visits per site; average proportion of fertilized pods per site; average number of seeds per pod. Models for richness, visits and seeds per pod had quasi-Poisson error structures to account for overdispersion, and the model for fertilized pods had a quasi-binomial error structure. Models for seeds and fertilized pods were based on average values per site and were weighted with variance per site to account for this [40].

To test community relationships between biodiversity and ecosystem function (question 2), we used bipartite network analysis (bipartite package [41]) in a novel way: to link pollinator communities to sites in a tropical agricultural landscape [22]. Based on traditional plant–pollinator networks, we replaced plants with habitats and constructed a weighted (total visits) bipartite network linking sites (lower level nodes) with flower visitor species observed at the site (higher level nodes). Because our flower visitor data are collected from one plant species (*B. rapa*) at every site, we control for differences in the relative attractiveness of the sampled plant community at each site; therefore, a site ‘node’ is representative of habitat attributes. For each site and flower visitor, we calculated the node specialization or discrimination index (*d′*). For sites, low *d′* near 0 indicates ‘generalist’ sites, which mostly attract flower visitors that are common across the landscape, and high *d′* close to 1 indicates ‘specialist’ sites, which mostly attract species that are not found at other sites [42]. Similarly for flower visitors, low *d′* means they are common across the landscape, and high *d′* means they are only found at one site. Unlike some other node metrics, *d′* corrects for relative abundance of interacting nodes and is robust to effects of matrix size [42,43]. In addition to the whole-landscape network, we also constructed individual networks for each land use to aid interpretation. We calculated ‘node strength’ for each pollinator species node in the networks to identify key nodes influencing network structure. For example, in this network, the strongest nodes indicate which flower visitors the sites depend on most [43].

Modularity is an important indicator of network complexity and may play a key role in network function; higher levels of modularity may increase the resistance of a network to disturbance [44–46]. Modularity itself is mostly useful when comparing multiple networks. However, the analysis also allows the identification of key nodes (in this case, sites or flower visitors) that influence the robustness and interaction structure of the network. We used the QuanBiMo algorithm, following the method and code described in [47], to calculate modularity (*Q*) for the landscape network and used the nullmodel function (100 randomizations; method ‘vaznull’) to convert *Q* to a *z*-score; a *z*-score above approximately 2 indicate the network is significantly more modular than random networks. We calculated participation coefficients (also called between-module connectivity, *c*-values or inter-module connectivity) and within-module degree (also called *z* values) for each site and flower visitor species within the network to identify which sites and flower visitor species were important connectors in the network. Participation coefficients can identify transition zones between environmentally different regions and may describe edges where taxa from different regions mix [48]. We used the null models (described above) to calculate recommended thresholds for participation coefficients and within-module degree for each network level, based on 95% confidence intervals (see electronic supplementary material for code). This method is recommended to objectively define critical thresholds for weighted networks [47]. We used these thresholds to identify network hubs (high participation coefficient, high within-module degree), between-module connecters (high participation coefficient, low within-module degree), within-module hubs (high within-module degree, low participation coefficient) and peripheral sites/species (low participation coefficient, low within-module degree) [22,44,47].

To determine whether land use influenced site node metrics, we used linear models to test for a significant difference among land uses for each site node metric (*d′* and participation coefficient). We also evaluated this using linear models for flower visitor *d′* (grouped by all species found in each land use); flower visitors with high *d′* are found at very few sites, while lower *d′* indicates generalist flower visitor species that are found across many sites within the landscape. To determine whether spatial proximity of sites influenced the similarity of node metrics, we calculated the distance (in kilometres) between each pair of sites and the difference between node metrics (participation coefficient and *d′*) for each pair of sites and used standard linear models to determine whether sites closer together in the landscape had more similar node metrics: response variables were Euclidean pairwise similarity between site node metrics and the predictor was pairwise metric distance between sites.

To determine whether the network influence of a site is associated with plant reproduction (question 3), we focused on two metrics for each site: the participation coefficient and discrimination index (*d′*). These metrics indicate an individual node's influence on the robustness of the functional network, as well as its role in connecting other nodes and modules across the landscape network. The *d′* index indicates a site's level of specialization (in terms of attracted flower visitors) relative to the rest of the network. We used GLMs to describe the relationship between plant reproduction (mean fertilized pods; mean number of seeds per pod) and the participation coefficient and *d′* for each site (*n* = 20). Fertilized pod (proportions) and seed data (counts) had high variance-to-mean ratios (seeds = 4.2; fertilized pods = 1.8), so we used a quasi-binomial error structure for fertilized pod models and quasi-Poisson for seed set models to account for overdispersion. Models were weighted with the variance of the response variable to account for variation in the data that would be masked by using average values for each site [40].

We then used a model selection procedure to explore whether node metrics (participation coefficients and *d′*) were influenced by pollinator community metrics or landscape composition. We calculated the proportion of each land use within 100 and 250 m buffers around each site in ArcGIS using the layer ‘Wet Tropics LU 2009’ (Department of Environment and Science, State of Queensland, 2018). These buffer distances were independent of each other (no overlap) and are representative of landscape influences on our focal taxa (insect pollinators) in the study region [49]. From this, we calculated the richness of different land use types within each buffer and the proportion of land use of the same type as the study site. We ran Gaussian GLMs to test the response of each node metric (participation coefficient, *d′*) to two sets of predictors: (i) pollinator community (total visits; species richness; Hymenoptera visits; Syrphidae visits; other Diptera visits; Coleoptera visits); and (ii) landscape composition (land use richness 100 m; land use richness 250 m; proportion of same land use 100 m; proportion of same land use 250 m). We used a model selection procedure (package MuMIn [50]) to identify the predictor from each set that explained most of the variation in each node metric. We used ΔAICc less than 2 to indicate the most explanatory models; if the intercept-only model was ranked the most appropriate, no subsequent models were considered suitable [51].

All analyses were conducted in R v. 3.5.0 (R Foundation, 2018) and PAST 3.15 [52].

## 3. Results

### (a) Effect of land use type on pollination services

**Figure 1.**
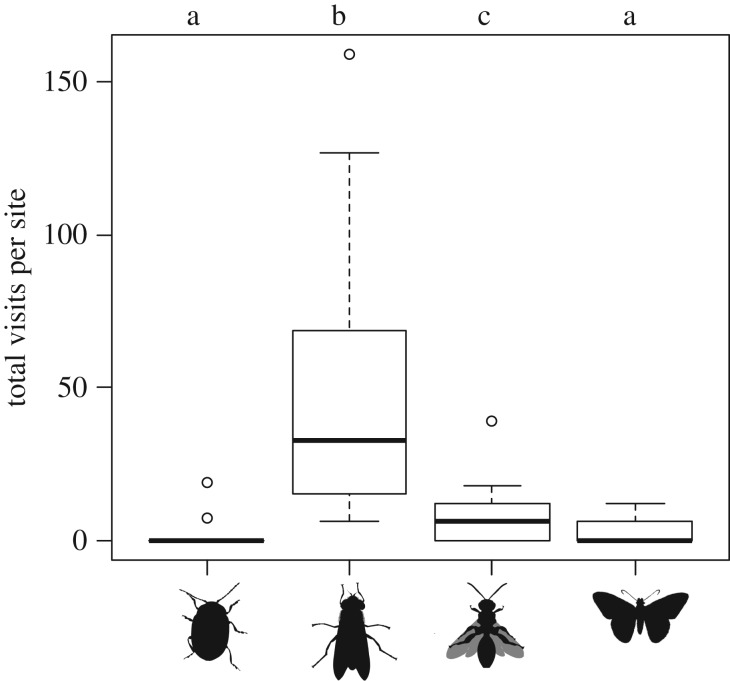
Total visits to flowering *B. rapa* pots by each taxonomic order (Coleoptera; Diptera; Hymenoptera; Lepidoptera). Letters above bars represent pairwise differences with other groups (*p* < 0.05). Edges are 25–75% intervals and whiskers show the inter-quartile range.

Community composition of taxonomic orders of flower visitors differed between land use types (total SS = 3.07, within-group SS = 1.97, *F* = 2.946, *p* = 0.02), but there was high overlap at the landscape scale (electronic supplementary material, figure S2). Diptera species were the most common flower visitors (*n* = 890) and were the only group recorded at every site (figure 1; electronic supplementary material, table S1). Almost half (48%) of all visits to sentinel flowering potted plants were by Syrphidae species (see electronic supplementary material). Hymenoptera were the second most common visitors (*n* = 158), and there was no difference between numbers of Lepidoptera (*n* = 60) and Coleoptera (*n* = 26) visitors (figure 1).

**Figure 2.**
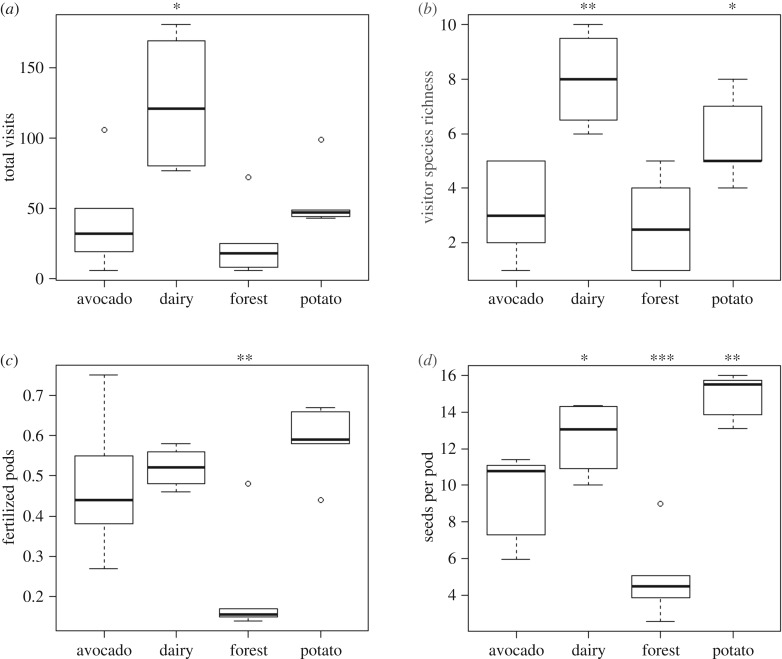
Effect of land use type on flower visitation and plant reproduction: (*a*) total visits by all flower visitors; (*b*) species richness of flower visitors; (*c*) proportion of fertilized pods; (*d*) number of seeds per pod. Asterisks above categories denote significance: *p < 0.05; **p < 0.01; ***p < 0.001.

All visitation and plant reproduction variables differed between land uses (figure 2; electronic supplementary material, table S2). Species richness and total number of visits of flower visitors were highest at dairy sites and lowest at forest sites (figure 2*a,b*; electronic supplementary material, table S2). Sentinel plant reproduction was highest at potato sites and lowest at forest sites (figure 2*c*,*d*). Syrphidae visits were also the strongest contributor to the number of fertilized pods, while visits by Syrphidae, Hymenoptera and Lepidoptera had the strongest effect on the number of seeds per pod (electronic supplementary material, figure S3).

### (b) Site–pollinator network structure and landscape effects

**Figure 3.**
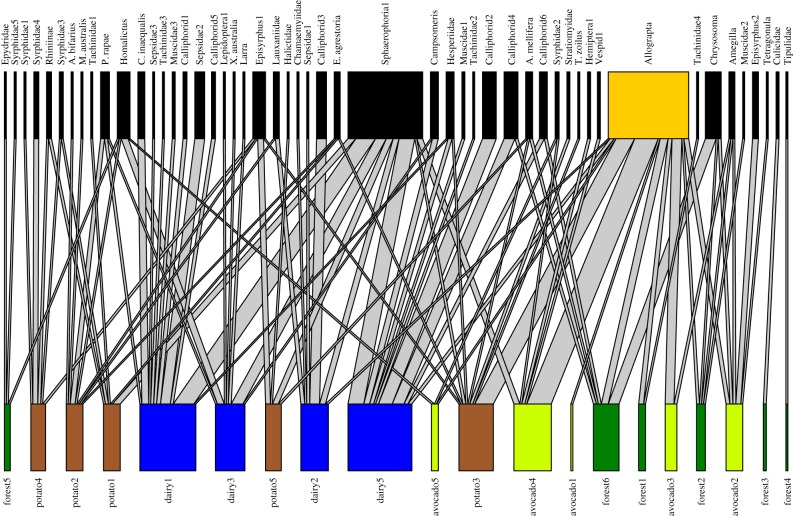
The bipartite site–pollinator landscape network. Sites are coloured by land use: dark green = forest; light green = avocado; blue = dairy; brown = potato. The yellow node in the top layer indicates the pollinator species with the highest node strength (*Allograpta* sp.). Width of grey connecting lines indicates weighted interactions. (Online version in colour.)

The landscape network (figure 3) was significantly modular (*Q* = 0.56, *z* = 4.39; electronic supplementary material, figure S4). Site node metrics did not differ between land use types: participation coefficient (*F* = 0.56_(3,16)_; *p* = 0.65) and *d′* (*F* = 2.54_(3,16)_; *p* = 0.09; electronic supplementary material, figure S5). There was a significant difference between land use types in the number of unique versus common species (flower visitor *d′*) recorded within each land use (*F* = 7.73_(3,65)_; *p* < 0.001). Sentinel pots at forest sites were visited by more unique species (i.e. species with higher *d′*) than sites in other land uses, and dairy sites had more visitors that were common across the network (electronic supplementary material, figure S6).

For our analysis of network hubs, we identified recommended critical thresholds for sites (participation coefficient greater than 0.60 and within-module degree greater than 1.88) and for flower visitors (participation coefficient greater than 0.57 and within-module degree greater than 3.12). No sites or flower visitors were considered network hubs (i.e. they did not exceed thresholds for both metrics; electronic supplementary material, figure S7). Four flower visitor species were important between-module connectors in the network (high participation coefficient, low within-module degree): Diptera: *Sphaerophoria* sp.; Hymenoptera: *Homalictus* sp. and *Apis mellifera*; Lepidoptera: Hesperiidae sp.

Distance between pairs of sites had a negative effect on the pairwise difference between each site's *d′* index (i.e. sites that were closer together were less similar in their level of visitor specialization compared with sites that were further apart; electronic supplementary material, table S3). This means that more specialized sites were scattered across the landscape, not aggregated in space. There was no effect of geographical distance between sites on similarity in participation coefficients (electronic supplementary material, figure S8).

### (c) Network structure and ecosystem function

*Allograpta* sp. (Diptera: Syrphidae) was the most influential flower visitor species (node strength = 4.94) in the landscape network (figure 3). The influence of Syrphidae was also apparent in each individual land use network: Syrphidae species were the strongest flower visitor node in each individual land use network: forest and avocado (*Allograpta* sp.), dairy and potato (*Sphaerophoria* sp.) (electronic supplementary material, figure S9).

Sites with higher participation coefficients had higher rates of plant reproduction, while more specialized sites (*d′*) had lower rates of plant reproduction (electronic supplementary material, figure S10). Hymenoptera visits were the best predictor for participation coefficients, but no community-level metric (i.e. species richness, total visits or visits per pollinator group) explained variation in *d′* (electronic supplementary material, table S4). Landscape composition within 100 m or 250 m did not affect either node metric (electronic supplementary material, table S5).

## 4. Discussion

Understanding which species are present in a given community and how they connect with other species and habitats is an important tool to understand ecosystem function in modified landscapes. This is because species respond differently to their habitats, and communities at selected sites within habitats are rarely uniform. Incorporating species identities and site level interactions acknowledges the relative contributions by different taxa to local communities, which is overlooked when using standard metrics such as species richness. In this study, a diverse insect community contributed to connections among nodes and plant reproduction. Species richness and total number of pollinator visits differed across land use types and Diptera, particularly generalist Syrphidae species, were the most frequent flower visitors across all land uses. Sentinel plant reproduction was highest in the more disturbed land uses (i.e. cultivated potato sites) and lowest at remnant forest sites. Syrphidae visitors were also the strongest contributor to the number of fertilized pods, while visits by Syrphidae, Hymenoptera and Lepidoptera had the strongest effect on the number of seeds per pod.

Importantly, we found that elements of network modularity (i.e. structural indicators of network connectivity in the landscape) had a positive influence on plant reproduction via the insect pollination of a sentinel plant. Participation coefficients were a useful predictor of a site's or flower visitor's influence on ecosystem function: sites with higher coefficients had higher rates of plant reproduction, and flower visitor taxa with high coefficients were identified as having the greatest effect on plant reproduction. While visits by Hymenoptera were the best predictor for participation coefficients, two syrphid species, *Allograpta* sp. and *Sphaerophoria* sp., were also identified as key nodes in the landscape-scale and land use networks. Yet participation coefficients were not significantly related to other community-level metrics, including total visits or species richness, nor to surrounding landscape composition. This suggests that the relationship between participation coefficients and plant reproduction is different to that of other standard community metrics and requires greater research attention. In other types of networks, higher participation coefficients have been associated with increased cognitive [53] and metabolic [54] function. Here, we show that participation coefficients may also be an indicator of ecological function in a heterogeneous landscape. Identifying highly connected flower visitor species that have a strong influence on network structure is a significant step forward to inform conservation priorities and decision-making in diverse agroecosystems. For example, the key pollinator taxa we identified here (*Allograpta* and *Sphaerophoria* spp.) are common pollinators for a wide range of plant families in Australia and globally [55–57], yet very little is known about their ecology and distribution in Australia. Steps to incorporate their resource and habitat needs into habitat and management plans are a critical next step to ensure their conservation.

Understanding the causes and consequences of modularity is important as it can have a positive effect on stability and ecosystem function [44,58,59], especially in human-dominated landscapes and disturbed systems [60]. Although this modularity–stability relationship is generally considered in terms of mutualistic plant–pollinator networks [44], future work should aim to determine how this translates to pollinator–habitat networks [22]. The landscape network in this study was significantly modular compared with a random network. While site node metrics did not differ between land use types, sentinel pots at forest sites were visited by more unique species (i.e. species with higher *d′*) than sites in other land uses, and dairy sites had more visitors that were common across the network. Modules comprising multiple sites from different land uses contained a diverse assemblage of flower visitors from different taxonomic groups, whereas five modules contained only one site (all forest or dairy sites), all of which were dominated by Diptera species. Single site modules thus had unique attributes compared to other sites in the network. For example, in these modules, we found predominantly calyptrate flies at dairy sites, and the rarely observed taxa (e.g. Culicidae, Epydridae) in the modules containing only a forest site.

These results suggest that modules in our network represent common niches [61,62] (i.e. matches between site attributes and flower visitor traits). This is further supported by the fact that the sites in each module did not appear to be grouped by geographical proximity (see electronic supplementary material, figure S1 for landscape map), and distance between sites had no effect on their similarity as module connectors. These results support recent studies that suggest that habitat heterogeneity (and temporal dynamics) is the most common cause of modules in field ecology networks [59]. Where landscape is considered, it appears that module structures may also be associated with major habitat divisions between structurally dissimilar habitats (e.g. freshwater and terrestrial, forest and cropland [46,63,64]). Hence, the dominance of calyptrate Diptera in modules dominated by dairy sites is not surprising, because Diptera are common in livestock systems, where they source multiple resources needed for their life cycle (e.g. carrion and dung [65,66]). Recent evidence shows pollinating Diptera taxa also rely on forest remnants in the same tropical landscape [49]; in our study, this is reflected in the diversity of Diptera species (especially Syrphidae) present in modules containing forest sites and the higher number of specialized Diptera species at forest that were absent in other land use types.

In our network, node specialization (*d′*) appeared to have a negative effect on ecosystem function. This effect was probably driven by the fact that two forest sites were the only sites with a *d′* index of 1 (highly specialized interactions), and visits by insects to sentinel pots were lowest in forest patches. Several dairy and potato sites also had high *d′*, because of a number of species at these sites that were not recorded anywhere else; yet these sites were also visited by other common insect taxa and had high rates of plant reproduction. Forest sites thus had the lowest rates of plant reproduction out of all land uses. This result, however, does not indicate that forest fragments are not contributing to ecosystem function in this landscape as we did not specifically test how habitat quality attributes (e.g. nesting sites, non-floral resources, environmental conditions) influence site specialization and only two of the forest sites appeared to be highly specialized in interactions. Rather, the variation across forest sites is probably due to our *a priori* selection of categorical land use types that attempted to standardize local habitat conditions but could not control for the influence of the surrounding matrix at greater spatial scales. Our landscape composition analysis showed that none of the landscape predictors (land use richness and heterogeneity within 100 and 250 m) had an effect on site node metrics—this may because the database layer used to measure land use was too coarse, or other local or landscape factors influenced site-level variations, such as the quality of nesting sites, non-floral resources or other environmental conditions. In order to more accurately assess how habitat quality attributes influence site specialization, future work would benefit from collecting information on structural attributes of individual sites. For example, we found greater overlap in community composition between forest and avocado sites compared to dairy or potato. This is likely to be because perennial tree crop orchards are more similar to the local forest in terms of physical structure and temporal dynamics, compared to open grassland or rotational crop systems [67].

Further, because the sentinel plant used is an exotic fast-growing annual plant that is more commonly found in open disturbed systems compared with clearings in unmanaged tropical rainforest [36,68,69], it is difficult to tease apart whether interactions between generalist pollinators and a generalist model crop plant increased pollination services in habitats suitable to the pollinators it interacts with or was a function of the sentinel plant selected. Our sentinel plant standardized the local floral resources available to local flower visiting insects and enabled us to compare visitation to the same plant across different sites and land use types without the biases of differences in floral structure, nectar and other resources. Selecting a single focal plant as a sentinel species therefore has its limitations, and future studies could attempt to use multiple plant species with different floral traits and habitat needs to tease apart habitat effects from the traits of the particular sentinel plant selected.

We have shown that network analysis can be applied in novel ways to understand important links between biodiversity and ecosystem function. By linking pollinator communities to sites in an agricultural mosaic landscape, using sentinel pots of a single plant species, we have shown how diverse groups of pollinators across the landscape contribute to plant reproduction in multiple habitat types. This approach has great potential to be applied in other systems, with valuable outcomes for conservation and management of ecosystem services in agricultural landscapes. An important next step is to increase knowledge on what aspects of landscape composition and habitat structure influence a site's participation coefficient, as this may enable land managers to adopt practices that support network connectivity.

## Supplementary Material

Supplementary Figures & Tables

## Data accessibility

All relevant data and R code are published on github: https://github.com/manusaunders/landusenetworks.
