# Supplementary material for: Network modularity influences plant reproduction in a mosaic tropical agroecosystem
Source: Proc Biol Sci. 2019 Mar 27;286(1899):20190296. doi: 10.1098/rspb.2019.0296 (PMC6452072; doi:10.1098/rspb.2019.0296)
Supplement: Supplementary Figures & Tables [file rspb20190296supp1.pdf]

Electronic Supplementary Material

Article: Network modularity influences plant reproduction in a mosaic tropical agroecosystem

Authors: Manu E. Saunders & Romina Rader

Journal: Proceedings of the Royal Society B

DOI: 10.1098/rspb.2019.0296

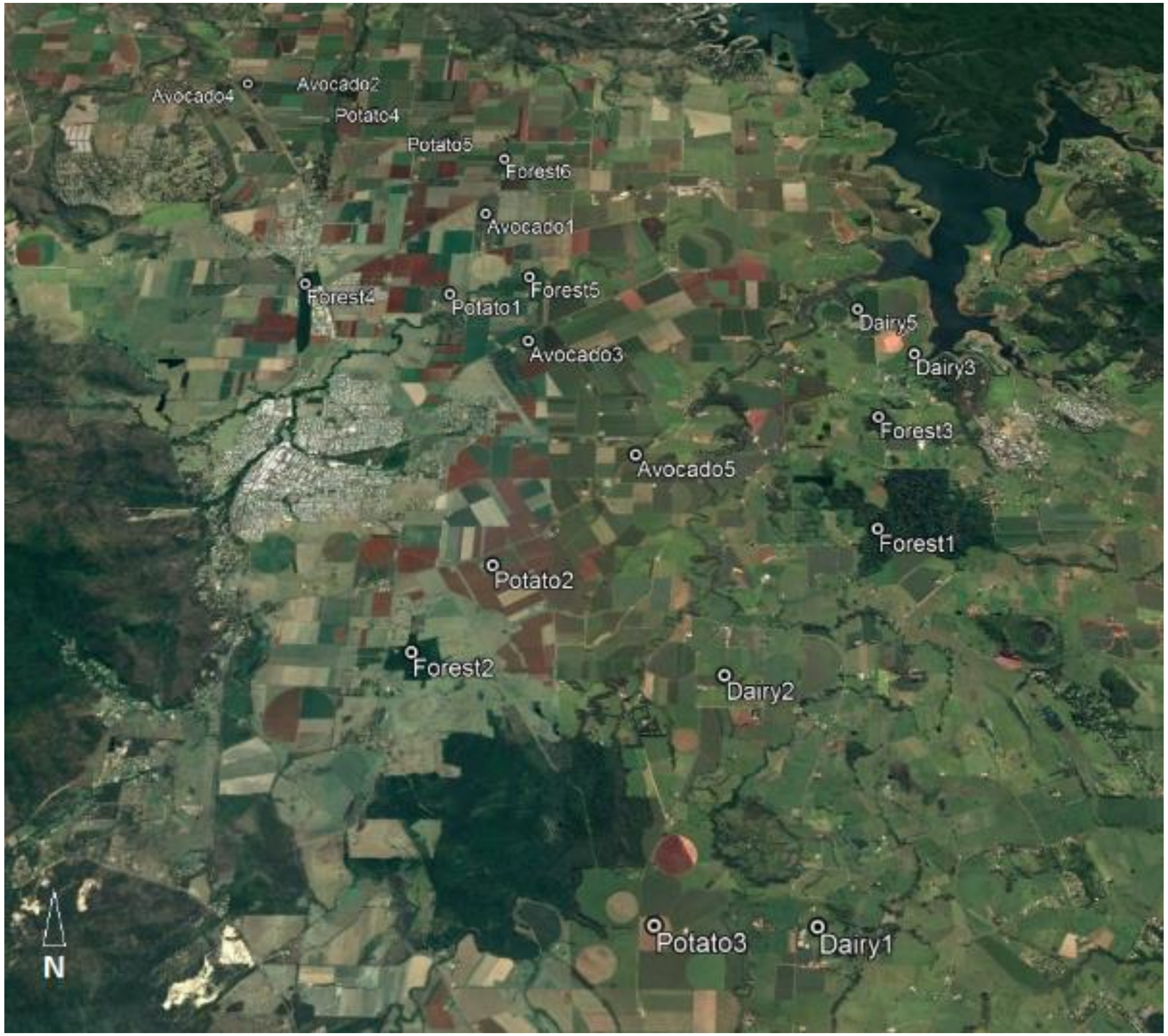

Figure S1. Illustrative map of relative locations of study sites. Source: Google Earth Pro, eye altitude 20.02 km.

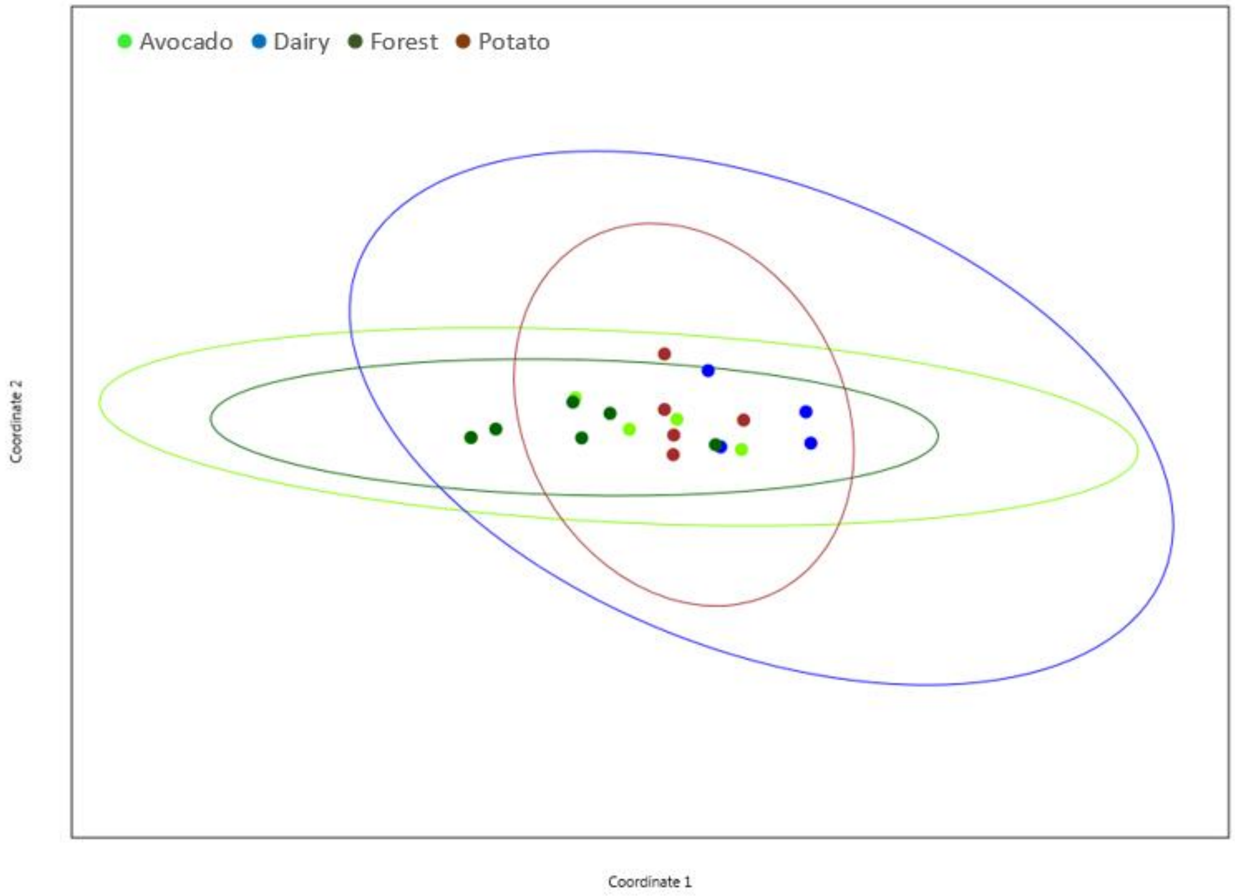

Figure S2. Community composition of flower visitor communities at each site within the four land uses (NMDS plot including 95% ellipses; Stress = 0.06).

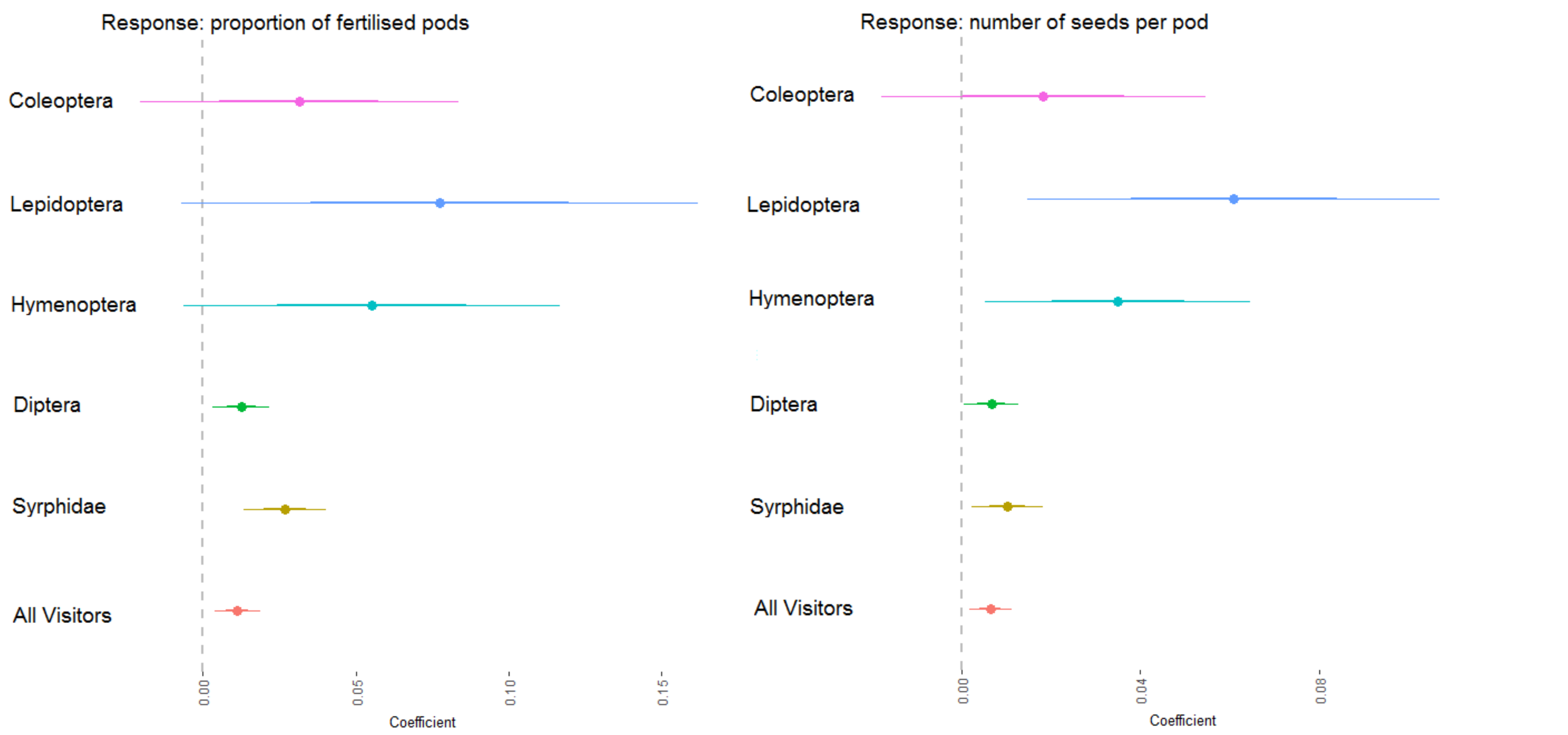

Figure S3. Effect of visitation by each taxonomic group on plant reproduction.

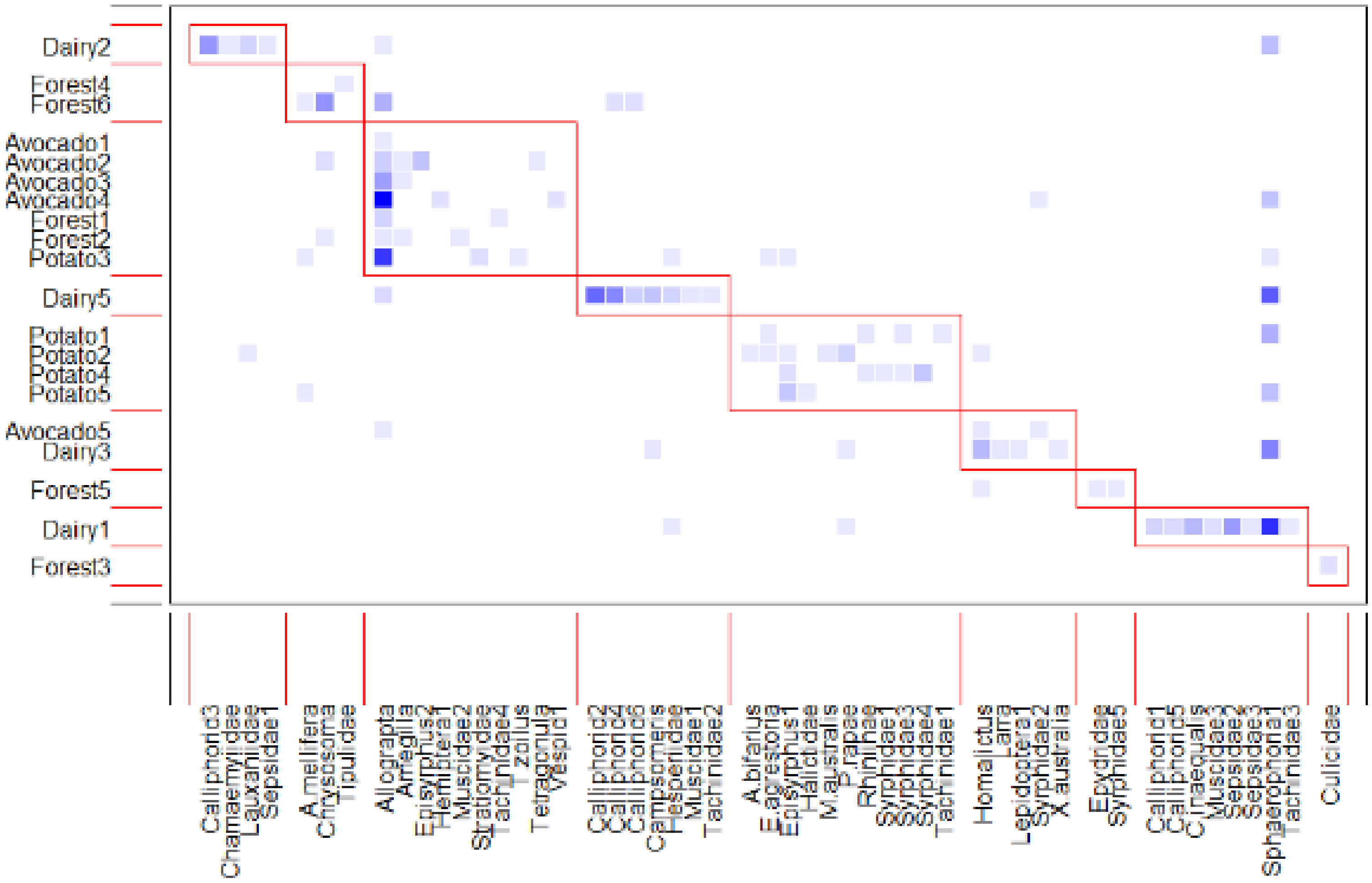

Figure S4. Modularity plot for the landscape network

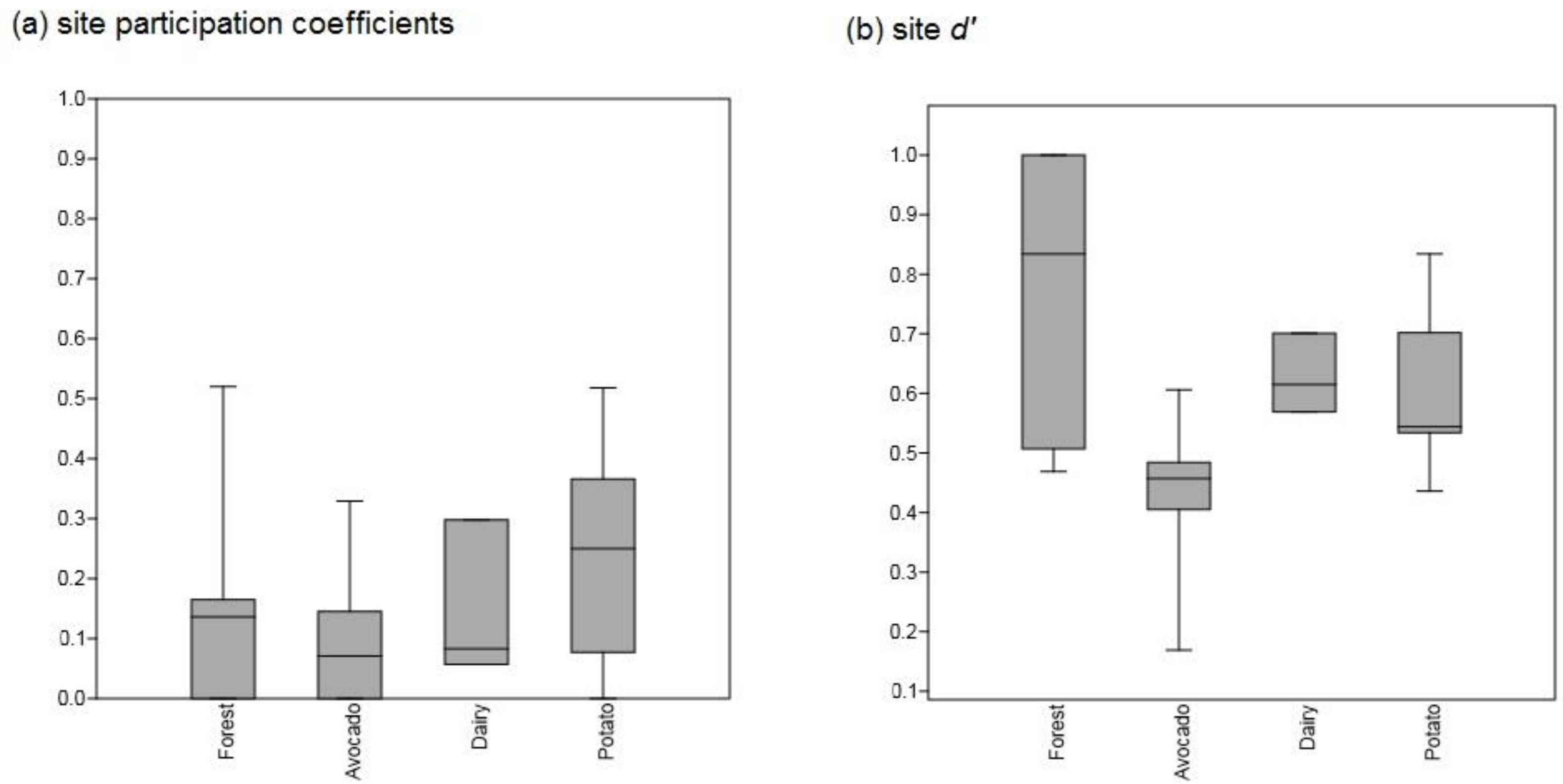

Figure S5. Median site metrics for each land use type

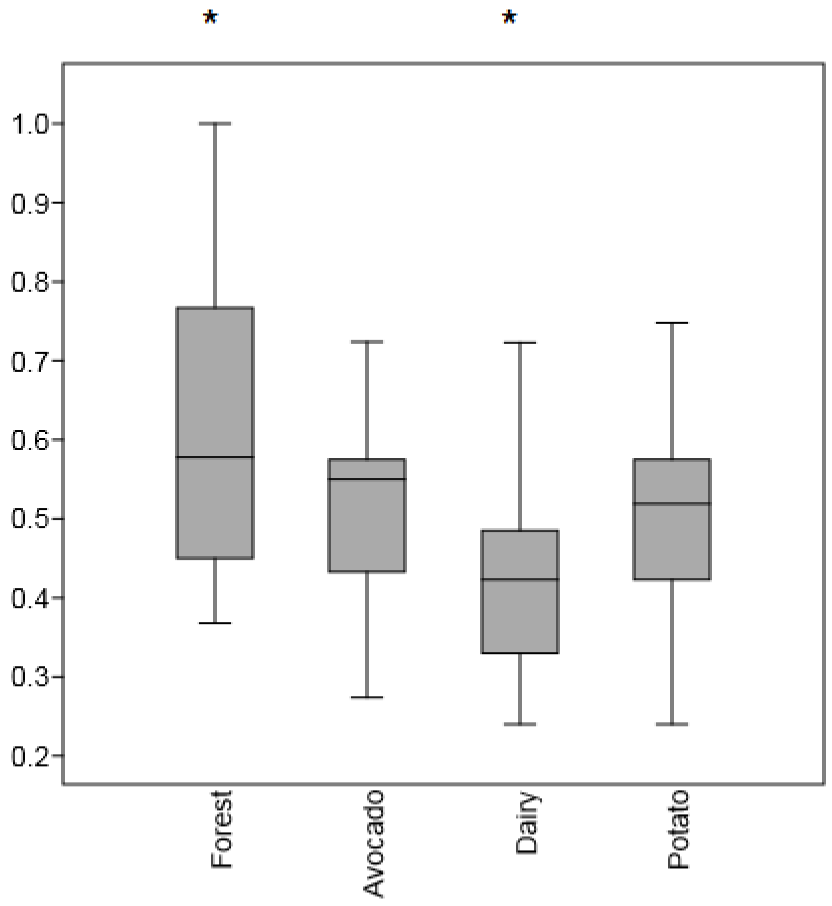

Figure S6. Specialisation (d') of all flower visitor species found in each land use type. Asterisks denote significance: *p < 0.05

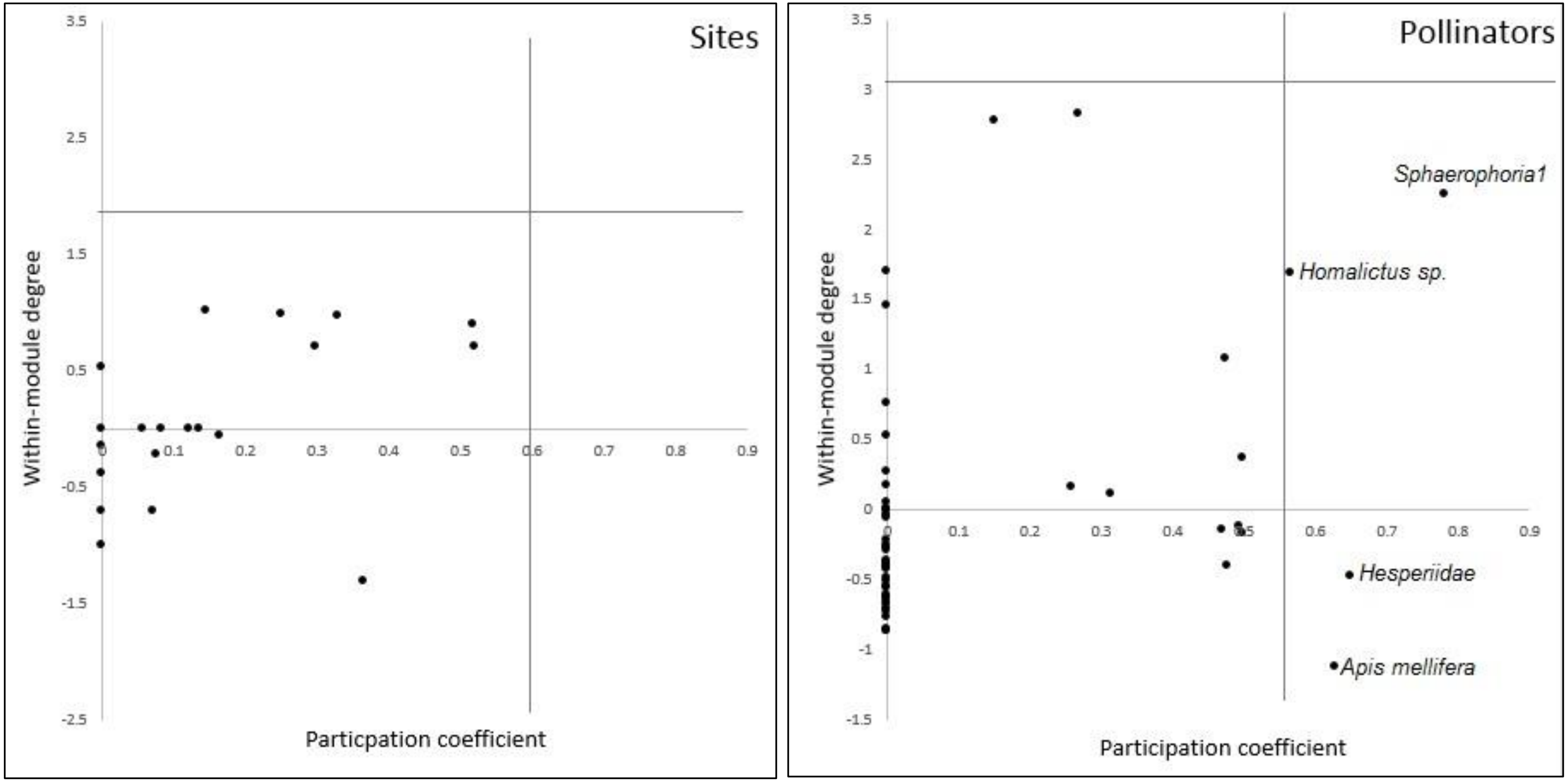

Figure S7. c-z correlations identifying key connector nodes. Interior lines show the critical thresholds for each metric. We only identified flower visitor nodes as between-module connectors in our network (nodes in the bottom right quadrant).

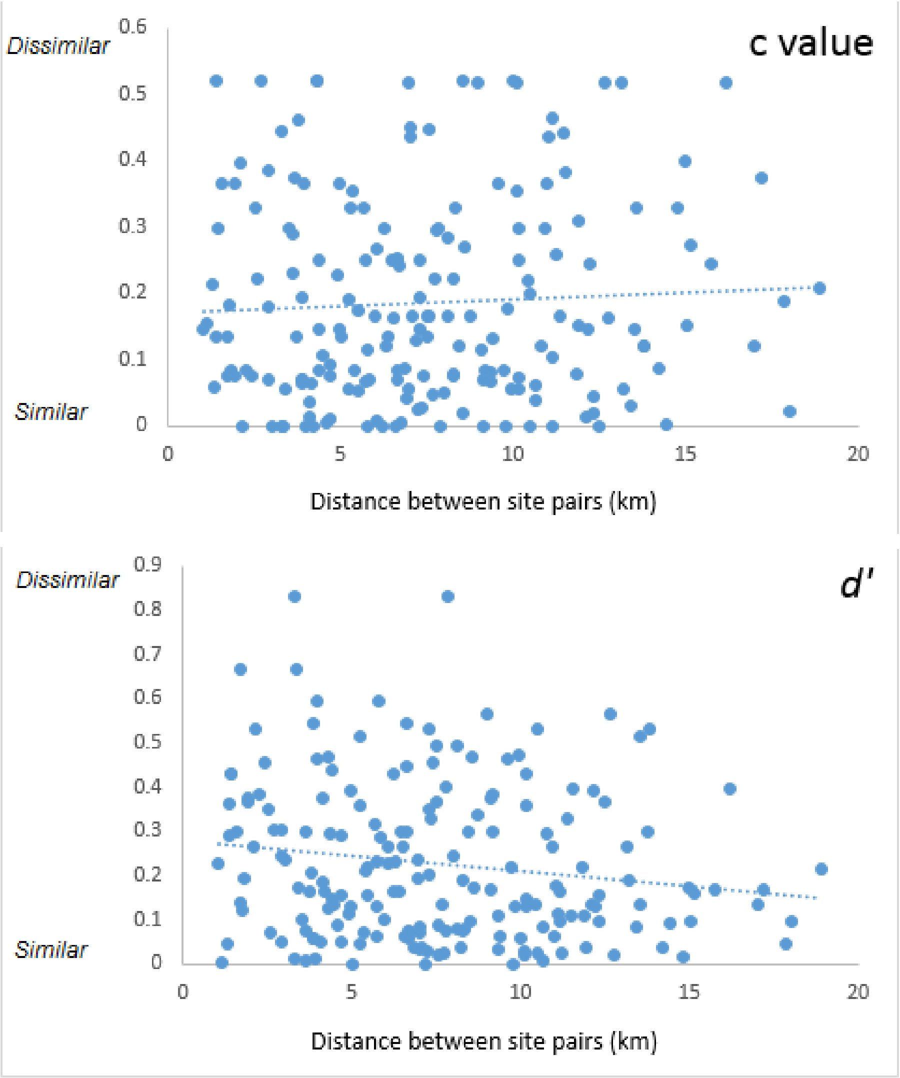

Figure S8. Pairwise relationships between geographical distance and difference in node metrics for all site pairs.

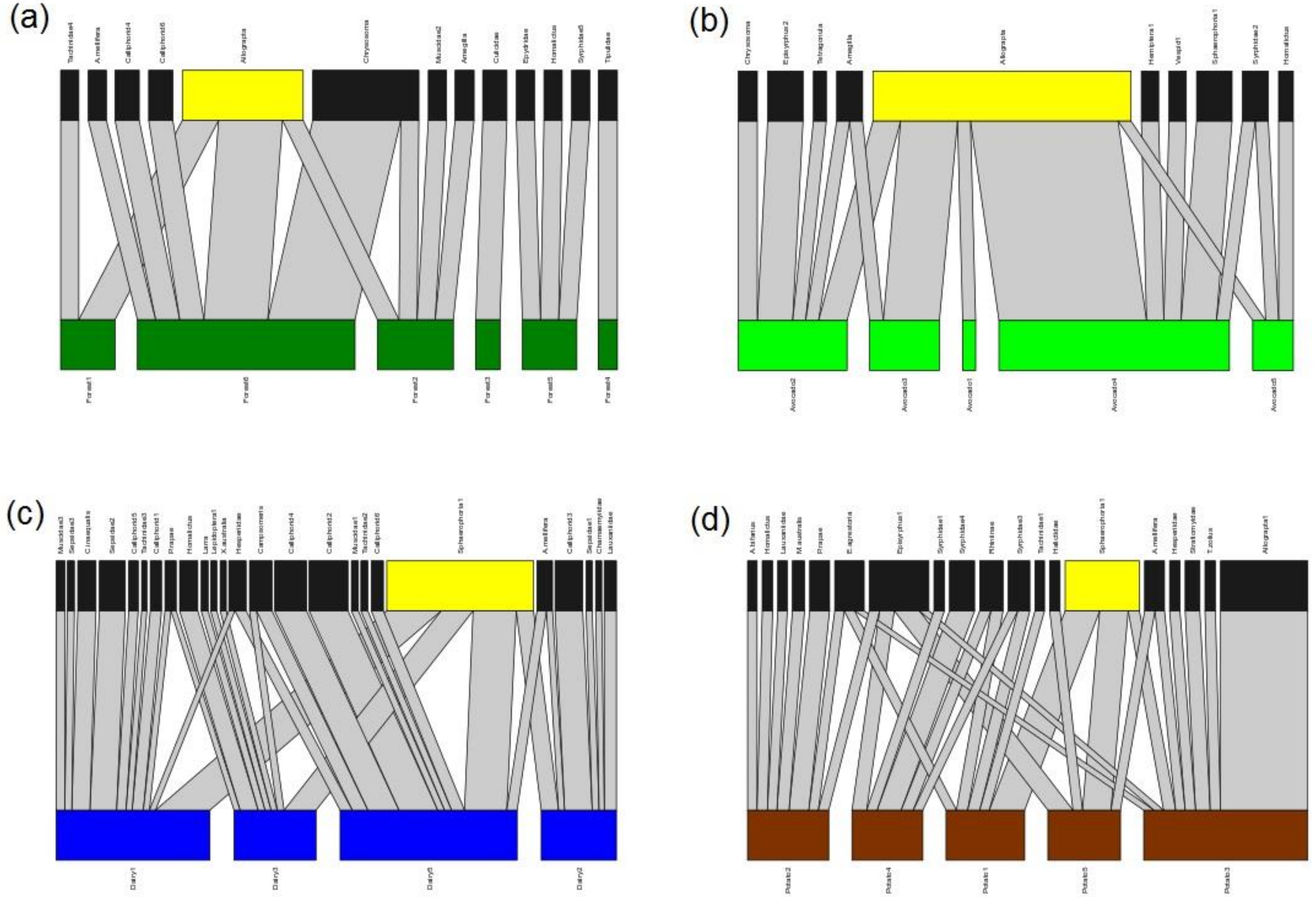

Figure S9. Individual land use networks for (a) forest, (b) avocado, (c) dairy, (d) potato. Yellow nodes on the top level indicate the pollinator species with the highest node strength for that land use: (a) and (b) Allograpta sp.; (c) and (d) Sphaerophoria sp.

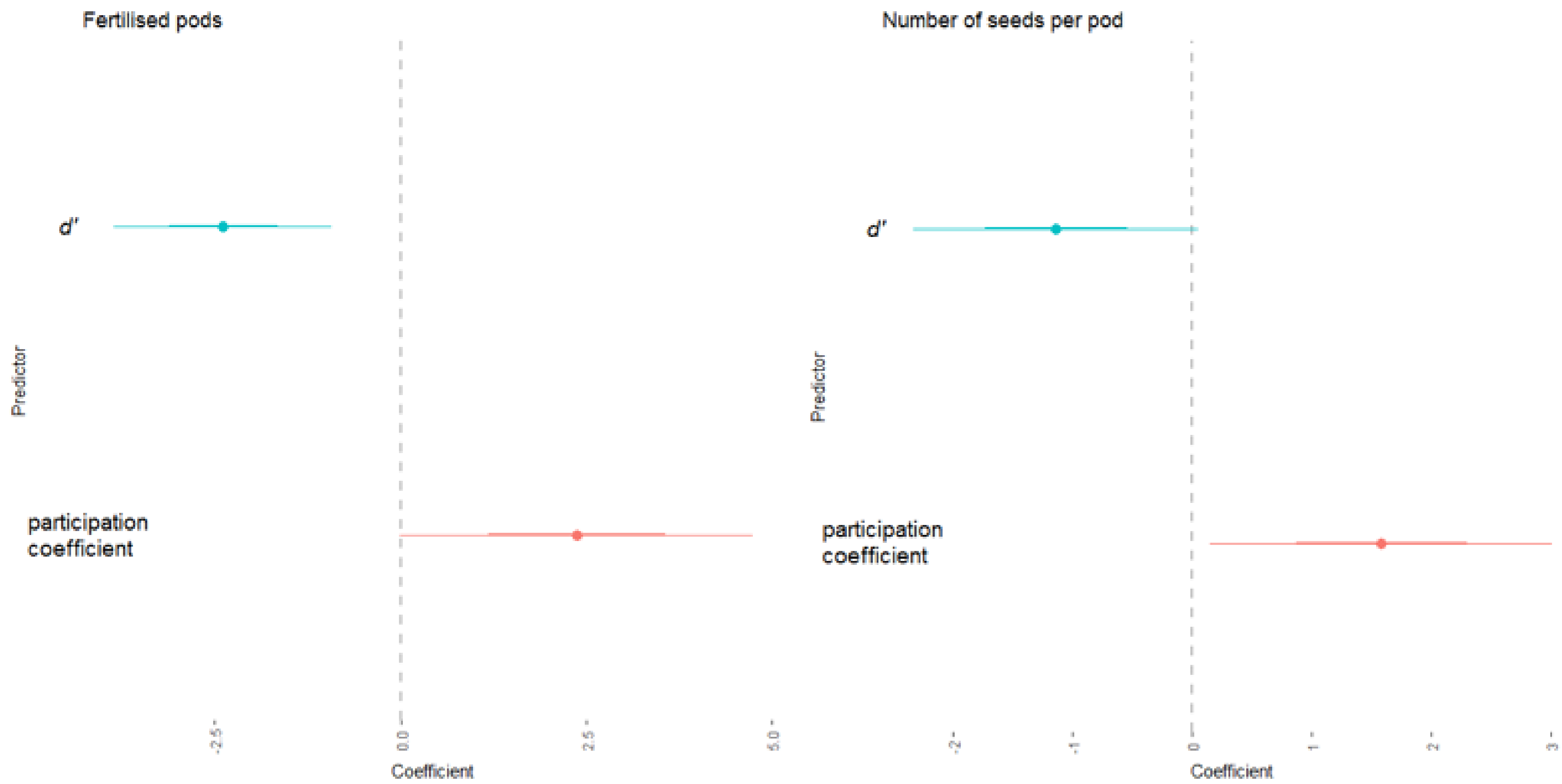

Figure S10. Relationships between node metrics and plant reproduction at each site.

Table S1. Estimates for glm(Visits~Order). Coleoptera as reference category

Predictor	Est ± SD	t value	95% Confidence Intervals
Diptera	3.53 ± 0.85	4.12	2.20, 5.80
Hymenoptera	1.81 ± 0.91	1.99	0.29, 4.13
Lepidoptera	0.84 ± 1.01	0.83	-1.03, 3.26

Table S2. Estimates for glm(response~Land Use). Avocado as reference category

Response	Land Use	Est ± SD	t value	95% Confidence Intervals
Total visits	Dairy	1.08 ± 0.39	2.74	0.34, 1.90
	Forest	-0.55 ± 0.52	1.07	-1.62, 0.45
	Potato	0.281 ± 0.44	0.64	0.07, 1.14
Visitor species richness	Dairy	0.92 ± 0.27	3.44	0.41, 1.46
	Forest	-0.18 ± 0.31	-0.59	-0.79, 0.43
	Potato	0.60 ± 0.27	2.20	0.07, 1.14
Average proportion of fertilised pods	Dairy	0.22 ± 0.43	0.52	-0.62, 1.08
	Forest	-1.39 ± 0.37	-3.84	-2.11, -0.68
	Potato	0.44 ± 0.43	1.03	-0.39, 1.28
Average seeds per pod	Dairy	0.40 ± 0.20	2.06	0.02, 0.79
	Forest	-0.73 ± 0.18	-4.06	-1.09, -0.38
	Potato	0.55 ± 0.17	3.19	0.22, 0.90

Table S3. Estimates for lm(response~distance)

Response	Distance (Est ± SD)	Adj. R-squared	$F_{(df)}$	95% Confidence Intervals
Participation coefficient	0.002 ± 0.003	-0.003	0.521 (1,188)	-0.003, 0.007
d' (specialisation)	-0.007 ± 0.003	0.019	4.593 (1,188)	-0.013, -0.001

Response = pairwise Euclidean dissimilarity between metrics per site

Distance = pairwise geographical distance between sites

Table S4. Model selection results for effects of pollinator community metrics on node metrics.

Response	(Intrc)	AllVisits	Richness	Other Diptera	Hymenoptera	Lepidoptera	Coleoptera	Syrphidae	df	logLik	AICc	delta	weight
Participation Coefficient	0.0793				0.01241				3	11.474	-15.4	0	0.531
	0.02347		0.02868						3	9.929	-12.4	3.09	0.113
	0.07112							0.003152	3	9.907	-12.3	3.13	0.111
	0.1219						0.009191		3	9.752	-12	3.44	0.095
	0.1568								2	7.686	-10.7	4.78	0.049
	0.08727	0.001219							3	9.013	-10.5	4.92	0.045
	0.1244					0.01082			3	8.924	-10.3	5.1	0.041
	0.1685			-0.0007485					3	7.829	-8.2	7.29	0.014
d'	0.602								2	4.114	-3.5	0	0.32
	0.6569							-0.002019	3	4.703	-1.9	1.62	0.143
	0.6341				-0.005137				3	4.499	-1.5	2.02	0.116
	0.5843			0.001134					3	4.343	-1.2	2.34	0.1
	0.6183	-0.0002866							3	4.162	-0.8	2.7	0.083
	0.6107		-0.00188						3	4.12	-0.7	2.78	0.08
	0.6002						0.0004684		3	4.117	-0.7	2.79	0.079
	0.6011					0.0002928			3	4.115	-0.7	2.79	0.079

Table S5. Model selection results for effects of landscape composition metrics on node metrics.

Response	(Intrc)	Same LU 250m	Same LU 100m	LU richness 250m	LU richness 100m	df	logLik	AICc	delta	weight
Participation coefficient	0.1568					2	7.686	-10.7	0	0.472
	0.09585				0.03936	3	8.063	-8.6	2.04	0.17
	0.2034	-0.06232				3	7.738	-8	2.69	0.123
	0.1643			-0.002583		3	7.691	-7.9	2.79	0.117
	0.1586		-0.001839			3	7.686	-7.9	2.79	0.117
d'	0.602					2	4.114	-3.5	0	0.465
	0.3449		0.2755			3	4.389	-1.3	2.24	0.151
	0.5301			0.02478		3	4.385	-1.3	2.25	0.151
	0.6201				-0.01171	3	4.137	-0.8	2.75	0.118
	0.5889	0.01745				3	4.117	-0.7	2.79	0.115
